# A First Case of Fluorescence Polarization Biosensor-Based Assay for Rapid Monitoring of Protein API Content in Tablet Dosage Forms: Detection of Lysozyme in Tablets

**DOI:** 10.3390/bios15110724

**Published:** 2025-11-01

**Authors:** Svetlana M. Filimonova, Ksenia S. Balyklova, Dmitry O. Zherdev, Sergei A. Eremin, Liliya I. Mukhametova, Vadim B. Krylov, Nikolay E. Nifantiev

**Affiliations:** 1 Institute of Pharmacy, Federal State Autonomus Educational Institution of Higher Education I.M. Sechenov First Moscow State Medical University of Ministry of Health of the Russian Federation (Sechenovskiy University), 119435 Moscow, Russia; filimonova.svetl@mail.ru (S.M.F.); balyklova_k_s@staff.sechenov.ru (K.S.B.); 2 Department of Chemistry, Lomonosov Moscow State University, 119991 Moscow, Russia; dmitryzher@yandex.ru (D.O.Z.); saeremin@gmail.com (S.A.E.); liliya106@mail.ru (L.I.M.); 3 Laboratory of Glycoconjugate Chemistry, N.D. Zelinsky Institute of Organic Chemistry, Russian Academy of Sciences, Leninsky Prospect 47, 119991 Moscow, Russia; v_krylov@ioc.ac.ru

**Keywords:** lysozyme, fluorescence polarization analysis, biosensor, chitooligosaccharide, tablets quality control

## Abstract

Protein-based APIs represent a big group of modern therapeutics. Their characterization involves complex analytical protocols which require special methods, especially in the case when the protein drug is included into tablet dosage forms. Although the fluorescence polarization assay (FPA) is not currently regulated by many national Pharmacopeias, it represents a promising approach for protein drug standardization, considering their rapid, sensitive, and automatable detection suitable for high-throughput analysis and real-time quality control. To evaluate the applicability of FPA for the analysis of protein drugs in tablets, the quantifying of lysozyme in tablet dosage forms was studied by this method with the use of a fluorescently labeled synthetic chitooligosaccharide tracer. It was shown that this approach overcomes the limitations of the conventional turbidimetric assay of lysozyme determination, which is labor-intensive and relies on unstable reagents. Measurements were performed with both portable and stationary fluorescence polarization readers. Commercial tablets from five manufacturers containing lysozyme (20 mg) and pyridoxine hydrochloride (10 mg) together with other excipients were analyzed. The FPIA method showed a linear range of 5.0–70 µg/mL, with specificity confirmed by the absence of interference from excipients. Accuracy, evaluated by standard addition (10–20 mg), yielded recoveries of 100.2–106.0%. Placebo spiked with lysozyme at 80–120% of nominal content demonstrated recoveries of 98.0–100.1%, with RSD (*n* = 6) not exceeding 13.7%, indicating good precision. The developed method enables reliable lysozyme quantification in tablets, offering speed, simplicity, and robustness, and shows its suitability for the routine quality control of protein-containing dosage forms including the enzyme ones.

## 1. Introduction

The therapeutic often called biologics or biopharmaceuticals, is part of a big group of modern advanced therapeutics [1]. Currently, monoclonal antibodies [2], hormones and growth factors, cytokines and interferons, enzymes and fusion protein are actively used as medicines [3]. Their high efficacy arises from strong target specificity and biocompatibility, as many are analogs of natural human proteins. Unlike small molecules, therapeutic proteins can replace deficient proteins, modulate immune responses, or catalyze biochemical reactions.

Unlike conventional low-molecular drugs, which are standardized by the mass of the active substance (API), enzymes and other biopharmaceuticals are characterized by specific activity. Such standardization involves selecting and validating analytical methods, defining assay conditions (temperature, pH, buffer), and employing reference standards [4,5]. The problems that arise in enzyme standardization are, firstly, the complexity of the analysis. For example, for some enzymes (acting on large, insoluble substrates such as cellulose or fibrin), it is difficult to develop a simple and reproducible analysis. The second problem arises due to the influence of the matrix, since the composition of the final dosage form (tablets, capsules, lyophilizate) includes excipients (fillers, stabilizers). They can interfere within the analysis and often require extraction of the target protein. One more limitation is connected with the stability protein API and standards, which can deteriorate over time and require periodic replacement and recalibration [4,5].

Lysozyme (muramidase (EC 3.2.1.17)) is an enzyme exhibiting pronounced antimicrobial activity [6]. While the primary effect of lysozyme is connected with its enzymatic activity directed to the hydrolysis of β-(1→4)-glycosidic bonds between N-acetylglucosamine units within the peptidoglycan of bacterial cell walls [7,8,9], some other biological effects were investigated [10,11]. Today, lysozyme is widely employed in clinical practice as a pharmacological agent for the treatment of dental and otorhinolaryngological diseases due to its demonstrated antibacterial and anti-inflammatory properties [6,7,8,12]. Orodispersible tablets containing 20 mg lysozyme hydrochloride and 10 mg pyridoxine hydrochloride are indicated for the treatment of infectious conditions of the oral cavity and pharynx [8,13].

Lysozyme activity is measured by observing the lysis peptidoglycan. The most common substrate is a suspension of the bacterium *Micrococcus lysodeikticus* (now reclassified as *Micrococcus luteus*), which has a cell wall rich in peptidoglycan [14,15]. This finding forms the basis for the principal method of lysozyme quantification, which relies on spectrophotometric measurement of the reduction in turbidity (optical density) of a suspension of these bacterial cells upon enzyme addition. The decrease in suspension turbidity directly correlates with microorganism lysis induced by lysozyme activity [16]. In spite of the widespread use of this method, it is characterized by significant limitations: high labor intensity, the requirement for both a lysozyme reference standard and lyophilized *M. lysodeikticus* biomass, instability of the prepared bacterial suspension, and considerable duration of the analysis. The enzymatic activity of lysozyme can also be accessed through turbidimetric [16,17] or fluorometric methods [9,16] in liquid phase assays.

For the quantitative determination of lysozyme, various methods have been proposed (Table 1): enzyme-linked immunosorbent assay (ELISA) [18], surface plasmon resonance (SPR) using a sensor functionalized with gold nanoparticles [19], and electrochemical nanosensors [20,21]. In addition, some other physicochemical methods for lysozyme determination have been suggested including those which apply the reversed-phase high-performance liquid chromatography [22]. The use of these methods is time-consuming and operationally complex. In some cases, expensive reagents and equipment are required, which limits the possibilities of real-time clinical monitoring as a diagnostic approach. In this regard, the development of a rapid, inexpensive, and easy-to-perform method for lysozyme determination remains relevant for modern precision medicine. However, unlike biological assays based on the evaluation of muramidase activity, chromatographic methods, ELISA, SPR and electrochemical biosensors detect lysozyme molecules through structural characteristics but cannot differentiate between the active native form and denatured or inactive conformations.

The turbidimetric method is widely used to determine lysozyme activity; however, the use of bacterium *Micrococcus lysodeikticus* makes it difficult to standardize the method. Fluorescence polarization analysis (FPA) is becoming an alternative to the classical turbidimetric analysis of lysozyme activity.

FPA is one of the most promising methods for the rapid and accurate detection of intermolecular interactions [23], including antigen–antibody binding [24] and enzyme–substrate recognition [25,26]. Fluorescence polarization analysis (FPA) is a detection method based on fluorescence measurement, widely used to study intermolecular interactions in solution. The method relies on the change in the rotational diffusion rate of a fluorescent molecule with changes in its size [27]. It is known that after irradiation with plane-polarized light, a substance can emit different fluorescence intensities in different planes. As the molecular volume of the substance increases, for example, upon binding to another molecule, its rotational diffusion rate slows, and the difference between the fluorescence intensities in the perpendicular $I_{⊥}$ and parallel $I_{∥}$ planes increase. The fluorescence polarization signal (FP) is calculated as

(1) $$mP=\frac{I_{∥}-I_{⊥}}{I_{∥}+I_{⊥}}*1000,$$

The FPA method offers several advantages for protein drug standardization: it is rapid, simple, and amenable to automation and high-throughput analysis. FPA provides high sensitivity at low substrate and protein (including enzymes) concentrations; its signal is robust to minor fluorescence fluctuations, enhancing accuracy; and it enables activity measurement in the presence of certain excipients, supporting real-time quality control. However, the FPA method has its limitations and it cannot be applied to any enzymes while the methods for determining the activity of enzymes catalyzing the hydrolysis of large molecules are known [28]. The viscosity and temperature of the solution greatly affect the rotation of the molecules. Thus, any changes in the composition of the buffer or the addition of components that change viscosity can distort the results [29].

**Table 1:** Methods for investigation of lysozyme concentration.

Method	Recognition Reagent	LOD	LOQ	Detection Range	Matrix	Ref
Turbidimetric	*Micrococcus lysodeikticus*	1.94 µg/mL	3.86 µg/mL		Buffer	[17]
Turbidimetric	*Micrococcus lysodeikticus*	Nd *	Nd	2.3–23.8 units/mL	Cell culture systems	[16]
Fluorescence technique	*Micrococcus lysodeikticus* labeled with fluorescein	Nd	Nd	0.47–5.28 units/mL		
ELISA	Purified rabbit polyclonal anti-chicken lysozyme antibody	0.264 µg/mL		0.38–4.8 µg/mL	Hen egg-white	[18]
Surface plasmon resonance (SPR) sensors modified with gold nanoparticles (AuNPs)	lysozyme-imprinted (Lyz-AuNP-MIP)	0.008 μg/mL	0.026 μg/mL	Nd	Artificial plasma, artificial urine, and artificial tears	[19]
Electrochemical aptasensor	Aptamer/amino-rGO/ionic liquid/amino-mesosilica nanoparticles	2.1 × 10^−3^ pM		10^−2^–2 × 10^5^ pM	Egg white, serum, tears, wine	[21]
Reversed-phase high-performance liquid chromatography	Reversed-phase polymeric column (PLRP-S 250 4.6 mm, 300 Å pore size, 5 μm particle size)	2.07 μg/g	4.13 μg/g	3.3–16.7 μg/g	Cheese	[22]
Fluorometric	Chitin azure	Nd	Nd	Nd	Blood sample	[9]
Colorimetric assay	β-D-galactosyl chitotetraose derivative [Gal(GlcN)_3_D]			2–31 μg	Buffer solution	[30]
FPA	Fluorescein isothiocyanate-labeled peptidoglycan	Nd	Nd	Nd	Rheumatoid synovial fluids	[31]
FPA	Fluorescein isothiocyanate-labeled chitooligosaccharide				tears	[32]

Nd *—not determined.

Although FPA is not currently regulated by many national Pharmacopeias, it represents a promising approach for protein drug standardization, providing their rapid, sensitive, and automatable detection suitable for high-throughput analysis and real-time quality control. Thus, in some cases, FPA is mentioned as an analytical method for detecting pharmaceuticals in human blood and urine, as well as for identifying toxicants in food products. Standardizing enzyme preparations used in medicine is a challenging task. In addition to lysozyme itself, tablets contain additional substances, which can affect enzyme activity and the results. We propose the use of FPA for the quantitative determination of enzyme preparations in tablet dosage forms and demonstrate the capabilities of the method using lysozyme as a model. FPA is a homogeneous assay that can quickly and accurately determine reagent concentrations. The FPA method for determining lysozyme activity is described, where a fluorescently labeled peptidoglycan is used as a substrate [31], which is isolated from the bacterial cell wall. However, peptidoglycan preparations obtained at different times may differ from each other and the analysis results will be difficult to compare. In a previous study [32], we have shown that the use of fluorescently labeled synthetic chitooligosaccharide tracer can solve this problem. Therefore, to assess the applicability of the FPA method for the analysis of lysozyme in tablet dosage forms, this method was studied using a fluorescently labeled synthetic chitooligosaccharide tracer.

## 2. Materials and Methods

### 2.1. Reagents

A reference standard of hen egg-white lysozyme (activity ≥15,000 U/mg, Sisco Research Laboratory, Mumbai, India) was used. The spacer-armed chitotrioside was chemically synthesized from a glucosamine precursor and subsequently conjugated with fluorescein isocyanate as previously described [32]. All reagents were of analytical grade. Orodispersible tablets containing lysozyme (20 mg) and pyridoxine hydrochloride (10 mg) from five manufacturers were purchased from local pharmacies.

### 2.2. Fluorescence Polarization Assay

Quantitative analyses were performed using both Sentry 200 (Ellie LLC, Germantown, WI, USA) and TDx (Abbott Diagnostics, North Chicago, Il, USA) fluorescence polarization analyzers with standardized optical parameters (λex/λem 485/535 nm) in 10 × 75 mm borosilicate glass reaction vessels. A working tracer solution was prepared by dissolving the chitooligosaccharide tracer in 10 mM phosphate buffer (pH 7.4) to achieve a fluorescence intensity 10-fold higher than the background level, corresponding to a concentration of 3 nM. Then, 50 μL of lysozyme solution was added to 950 μL of the working tracer solution. All measurements and manipulations were performed at room temperature (25 ± 2 °C). The mixture was vortexed, and the polarization value was measured. Each measurement was performed in triplicate.

### 2.3. Calibration Curve Construction

Standard solutions of lysozyme were prepared using 10 mM phosphate buffer (pH 7.4) as a solvent to achieve final concentrations of 10, 20, 30, 50, 70, 100, 150, 300, and 400 μg/mL in the test tubes. The dependence of fluorescence polarization values (mP) on lysozyme concentration was fitted by SigmaPlot 11 software (Systat Software Inc., Palo Alto, CA, USA).

### 2.4. Assessment of Method Statistical Characteristics

The method was characterized by its half-maximal inhibitory concentration (IC_50_), limit of detection (LOD), and working range. The LOD was set at a 10% signal decrease from the maximum, and the working range was established from 20% to 80% of the maximum signal amplitude.

### 2.5. Sample Analysis

All investigated samples were tablet formulations with an approximate mass of 200 mg, containing 20 mg of egg-white lysozyme and 10 mg of pyridoxine as active pharmaceutical ingredients (APIs), along with excipients. For analytical procedures, twenty pre-weighed tablets were pulverized into a homogeneous powder using a mortar and pestle, and a precisely weighed aliquot was quantitatively transferred into a 100 mL volumetric flask. The powder was dissolved in 50 mL of 10 mM phosphate-buffered solution (pH 7.4) using ultrasonic bath treatment (200 W temperature 25 ± 2 °C, dissolution time—until complete dissolution, approximately 5 min). The solution volume was subsequently adjusted to the mark with the same buffer. For clarification, 1 mL of aliquot was taken from the resulting solution and centrifuged at 3000× *g* for 5 min. A total of 50 μL of the clear supernatant was used for subsequent analysis. Quantitative determination of lysozyme was performed using a calibration curve. Each sample was analyzed in three independent replicates (*n* = 3). The lysozyme content in the tablet was determined as the average measurement value in samples weighing 200.0, 400.0, and 600.0 mg.

### 2.6. Validation of the Method

The analytical range of the method was defined as the concentration range that ensures quantitative determination of lysozyme in the studied tablet dosage form, while observing the established acceptance criteria for precision and accuracy. The specificity (selectivity) of the method was assessed by determining cross-reactivity and potential interference from the components of the dosage form. The effect of these substances on the determination of lysozyme was studied under conditions simulating sample analysis.

To assess the accuracy, the addition method was used: 10 mg and 20 mg of the lysozyme standard (50% and 100% of the expected content) were added to a 200.0 mg sample of powdered tablet mass. Then the determination was carried out according to the method. Each measurement was carried out three times.

In this way, the effect of the matrix on the determination of lysozyme in the dosage form was assessed. The model placebo mixture represented a composition of excipients and API pyridoxine hydrochloride used in the production of orodispersible lysozyme tablets and included lactose monohydrate (86.3%), pyridoxine hydrochloride (5.6%), tragacanth gum (5.5%), magnesium stearate (2.3%), sodium saccharinate (0.2%) and vanillin (0.1%). To prove the suitability of the method for the quantitative determination of lysozyme in a tablet dosage form, standard samples of lysozyme corresponding to 80%, 100% and 120% of the declared amount were added to a placebo sample weighing 180.0, 360.0 and 540.0 mg. The precision of the method was assessed under repeatability conditions. For this, a series of measurements of at least six samples from one manufacturer were carried out. The result was expressed as the relative standard deviation (RSD, %).

## 3. Results and Discussion

### 3.1. FPA-Based Lysozyme Assay

**Figure 1:**
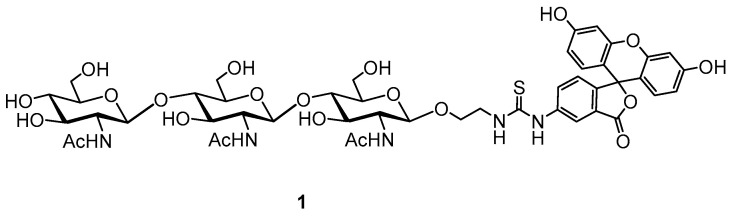
Structure of used oligosaccharide tracer **1**.

A new approach for lysozyme activity determination was proposed by using a fluorescence polarization assay (FPA), which was developed to study the activity of type-C lysozyme for diagnostic purposes, using fluorescently labeled natural peptidoglycan [28,31]. This inherent variability in natural peptidoglycan posed significant challenges in comparing lysozyme activity measurements across different experiments or laboratories, hindering reproducibility and standardization. The inconsistencies stemmed from variations in peptidoglycan structure and composition depending on bacterial source and extraction methods. These variations directly impacted the enzyme’s binding affinity and subsequent hydrolysis rate, leading to unreliable results. It is known that in addition to muramidase activity, lysozyme also has chitinase activity, therefore synthetic fluorescently labeled glycoconjugates—precisely defined, homogenous substrates—provide a much-needed standardized approach [32]. These synthetic substrates, chitooligosaccharides, including the chitotrisaccharide **1** (Figure 1) offer superior control over substrate composition, length, and labeling density. This ensures consistency in experimental conditions, minimizing the variability seen with natural peptidoglycans.

The refined FPA leverages these advancements. The assay begins by monitoring the increase in fluorescence polarization (FP) signal resulting from the rapid formation of a complex between the enzyme and the fluorescently labeled chitooligosaccharide which serves as a biosensor. This initial FP increase reflects the enzyme–substrate binding event. Crucially, the high specificity of this interaction ensures minimal interference from other proteins present in the sample.

Furthermore, the use of synthetic substrates allows for the precise quantification of enzyme activity using well-defined kinetic parameters, leading to significantly more reliable and comparable results across various experimental settings and laboratories. Advanced techniques, such as high-throughput screening adaptations of this FPA, are now being explored to expedite drug discovery and the development of novel lysozyme-based therapeutics. It is based on the use of a fluorescently labeled synthetic trisaccharide **1** tracer, which lysozyme specifically binds due to its chitinase activity. A comparative analysis of this FPA with the method based on the spectrophotometric monitoring of *M. lysodeikticus* suspension lysis demonstrated strong correlation between both methods, confirming that the binding of the synthetic substrate (chitotrisaccharide **1**) accurately correlates with the classical muramidase activity of the enzyme [33].

The FPA enables analysis with minimal analyte volumes, requires only minutes to perform, and is economically efficient. This method is employed for assessing food safety, monitoring environmental contaminants, detecting toxic substances [34], diagnosis infections [35,36], and analyzing enzymes and enzymatic activity [37,38]. The method principle is based on measuring the degree of depolarization of plane-polarized light induced by a low-molecular-weight substance conjugated with a fluorescent label. In its free state, this molecule exhibits high rotational diffusion rates, resulting in significant depolarization of emitted fluorescence and consequently yielding low fluorescence polarization (mP) values.

The development of efficient protocols for evaluation of the content of active pharmaceutical ingredients (API) in drug formulations including the tablets is a well-known analytical task [39,40,41]. They need the development of special approaches because of possible effect components being present in medicine formulations [41].

### 3.2. Optimization of the Method for the Quantitative Determination of Lysozyme in Tablets

**Figure 2:**
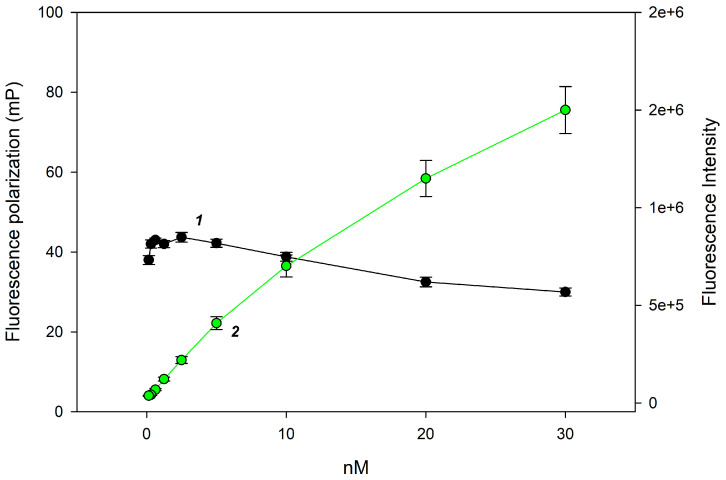
Changes in fluorescence polarization (1) and intensity (2) signals with the concentration of chitotrisaccharide **1** at 25 °C and pH 8.5.

The quantitative analysis of lysozyme in tablet dosage forms requires rapid and cost-effective methods. A promising approach is the use of FPA using a synthetic trisaccharide substrate **1**. The matrix of orodispersible lysozyme tablets is quite complex: it can include lactose monohydrate, pyridoxine hydrochloride, gum tragacanth, magnesium stearate, sodium saccharinate, etc. The dissolution of the tablet can result in a turbid solution, so we needed to adjust the tracer concentration to perform the analysis in colored and opalescent solutions. Therefore, we measured the change in fluorescence intensity and polarization as a function of the concentration of chitotrisaccharide **1** and selected a concentration range so that the fluorescence signal remained stable with a linear change in fluorescence intensity (Figure 2).

These conditions correspond to a chitotrisaccharide **1** concentration range of 1 to 5 nM. To ensure that turbidity or color of the solution does not affect the FP signal value, we chose a concentration of 2 nM.

**Figure 3:**
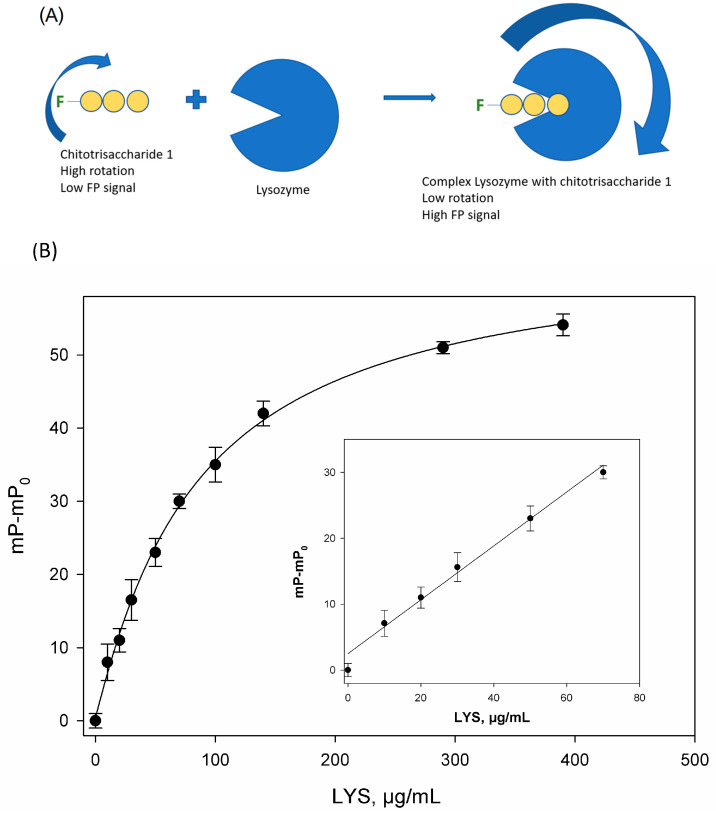
(**A**) Binding lysozyme to chitotrisaccharide **1**. (**B**) The change in fluorescence polarization signal (mP-mP_0_) versus lysozyme concentration in solution, where mP is the measured FP signal, and mP_0_ is the polarization of the free tracer. *n* = 3. pH 7.4; 25 °C. The inset shows the linear range of this curve.

The previous studies demonstrated that hen egg lysozyme selectively binds to chitotrisaccharide **1** without its degradation [32], which significantly accelerates the experimental procedure and simplifies data analysis. The observed increase in fluorescence polarization signal was attributed to the elevated molecular size of the fluorescently labeled substrate upon complex formation (Figure 3A). The changes in the fluorescence polarization signal (mP−mP_0_) at the different concentration of standard of lysozyme in the phosphate-buffered solution (pH = 7.4) is shown in Figure 3B. The change in the FP signal was dose-dependent and can be used for the quantitative determination of lysozyme in dosage forms.

The analytical characteristics of FPA were as follows: the detection limit (LOD) was 4.5 µg/mL, and the linear range was 5.0–70 µg/mL. Using this dependence of the FP signal on the lysozyme concentration, we can determine the unknown concentration of the active protein.

### 3.3. Results of Quantitative Determination of Lysozyme Content in Tablet Forms

Orodispersible lysozyme tablets containing 20 mg of lysozyme and 10 mg of pyridoxine were purchased from a local pharmacy. In addition to the active pharmaceutical ingredients (APIs), the tablets can contain lactose monohydrate, tragacanth gum, magnesium stearate, sodium saccharinate, and vanillin. Each tablet had a total weight of 200 mg. After grinding, portions of 200.0, 400.0, and 600.0 mg were weighed and dissolved in phosphate-saline buffer (pH 7.4) using ultrasonic bath treatment. Then 1 mL aliquots were taken from the resulting solution and centrifuged at 3000× *g* for 5 min and 50 μL of the clear supernatant was used for subsequent analysis. Quantitative determination of lysozyme was performed using a calibration curve presented in Figure 3B. Each sample was analyzed in three independent replicates (*n* = 3). It was found that the most accurate and convergent results were provided by samples weighing 200.0, 400.0 and 600.0 mg, which correspond to a concentration of 20, 40, and 60 μg/mL in the final solution (Table 2). The lysozyme content per tablet was determined as

(2) $$m\left(lysozyme\;in\;tablet\right)=\frac{m\left(\mathrm{lysozyme}\;\mathrm{from}\;\mathrm{calibration}\;\mathrm{curve}\right)*10^{3}*\mathrm{average}\;\mathrm{mass}\;\mathrm{of}\;20\;\mathrm{tablets}}{m\left(\mathrm{sample}\;\mathrm{tablet}\;\mathrm{wieght}\right)}$$

The use of samples weighing less than 150.0 mg resulted in a decrease in the precision of the analysis. The use of samples weighing more than 650.0 mg resulted in a decrease in the analytical signal due to the possible influence of excipients. Since the graph of the dependence of the analytical signal (mP) on the concentration of lysozyme in the analyzed solution has a sigmoidal shape, which can complicate the interpretation of the analysis, a linear fragment of the graph was taken for the quantitative determination of lysozyme in the dosage form (Figure 3B).

### 3.4. Validation of the Method

The analytical range of the FPA for the quantitative determination of lysozyme in tablet dosage form was 5.0–70 µg/mL. However, the determination of lysozyme in tablet samples over 650 mg, which corresponds to a lysozyme content of 65 µg/mL in the analyzed sample, is difficult due to the possible influence of excipients. Therefore, the analytical range for the method for the quantitative determination of lysozyme in tablet dosage form was reduced to 18.5–65 µg/mL. Tablet powder samples weighing 200 mg, 400 mg, and 600 mg were used for the analysis after appropriate dissolution and dilution.

Since the principle of the FPA method in this case is based on the enzymatic activity of lysozyme (direct binding to the substrate analog), in order to ensure accuracy and consider matrix effects, parallel determination was carried out in three independent weighed portions for each sample with subsequent calculation of the average value. For the analysis, tablet powders weighing 200 mg, 400 mg and 600 mg after appropriate dissolution and dilution were used. This approach allows us to conduct the following: to assess the effect of the dosage form matrix on the analysis, to check the constancy of the enzymatic activity of lysozyme at different dilution levels, and to select the optimal working dilution to minimize matrix interferences. The results of the parallel analysis of three weighed portions of tablet forms (Table 2) showed that all dilutions of the selected range give convergent results (*p* < 0.05); therefore, a weighed portion in the range from 200.0 to 600.0 mg can be used for the routine quantitative determination of lysozyme within the validated range. This method can also be employed to evaluate the content uniformity of the API. Given that the mass of an individual tablet is approximately 200.0 mg, a representative 200 mg aliquot of the homogenized tablet powder was used. The quantitative results obtained from this aliquot were consistent with data generated using other sample masses, confirming its suitability for homogeneity analysis.

To assess the specificity of the developed FPA method, a model mixture of a placebo composition was prepared, simulating the matrix of a tablet dosage form. Since the exact quantitative composition of commercial drugs is confidential, and the analyzed samples had a variable composition of excipients, the model mixture was compiled on the basis of the average composition characteristic of the studied dosage forms. Placebo samples of 180.0 mg, 360.0 mg, and 540.0 mg were analyzed under identical conditions as those used for the analysis of tablet samples. When analyzing the model placebo mixture, it was found that the analytical signal deviated from the solvent value by no more than 5.5%. Thus, there was no statistically significant cross-reactivity with pyridoxine hydrochloride and no interference from the components of the dosage form matrix.

The accuracy of the technique was proven by the addition method. An accurate weight of the standard lysozyme sample weighing 10.0 mg and 20.0 mg was added to an accurate weight of the powdered tablet mass of sample 1. Then the mixture was analyzed according to the developed technique. The study was carried out in three repetitions; the results are presented in Table 3.

Also, to assess the accuracy, model mixtures were analyzed consisting of a placebo with the addition of a sample of the lysozyme standard corresponding to 80, 100, and 120% of the declared content. For analysis, 200.0, 400.0, and 600.0 mg of the mixture were selected and analyzed under the same conditions as the tablet mass. Table 4 presents the results of the quantitative determination of lysozyme in the analyzed model mixtures.

Precision was determined by evaluating exact weighed portions of sample 1 weighing 200.0 mg, 400.0 mg and 600.0 mg and evaluating the RSD value, %. The results are presented in Table 5. The relative standard deviation RSD, % for all measurements did not exceed 13.7%.

### 3.5. Sample Analysis with the Abbott TDx Analyzer

**Figure 4:**
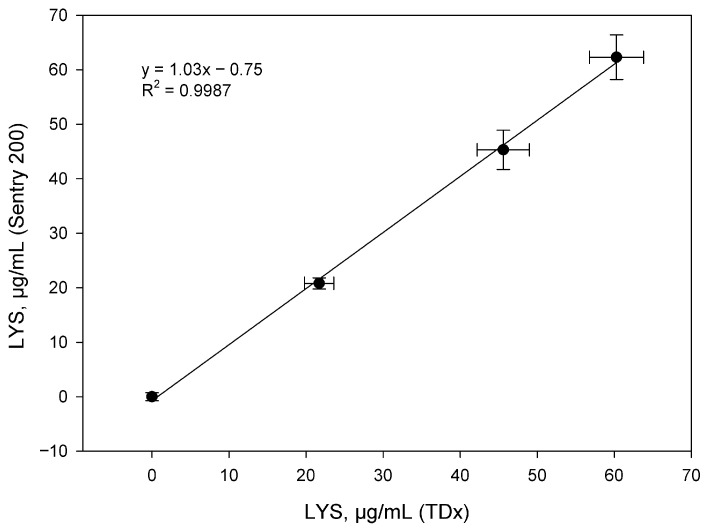
Correlation between LYS concentrations in 200.0, 400.0 and 600.0 mg tablet powders determined using the Sentry 200 and TDx instruments (n = 3).

The TDx analyzer is a stationary device with an automated system that provides parallel analysis of 10 samples, which significantly increases the productivity of routine studies. The work used identical procedures and reagent ratios previously tested for the Sentry 200 device. To construct a calibration curve, standard lysozyme solutions with concentrations of 10, 20, 40, 60 and 80 μg/mL were used. Parallel analysis of tablet form samples (sample 1) weighing 200.0, 400.0 and 600.0 mg was carried out. The results of the correlation of data obtained on both devices are presented in Figure 4.

Statistical analysis revealed a high correlation (R^2^ > 0.99) between the two systems, confirming their analytical equivalence for the quantitative determination of lysozyme in tablet dosage forms.

## 4. Conclusions

The described results show that the FPA may be used for controlling the quantity of lysozyme in tablet dosage forms. Using fluorescently labeled trisaccharide as a tracer has significant advantages over traditional methods, such as turbidimetry, by providing enhanced sensitivity and reduced susceptibility to excipients commonly found in tablets. Optimal sample weights for accurate analysis were determined to be 200.0 mg, 400.0 mg, and 600.0 mg, allowing for flexibility based on the expected lysozyme concentration. The method’s specificity was confirmed by demonstrating the absence of cross-reactivity with common tablet excipients, including binders, fillers, and disintegrates, ensuring that the measured fluorescence polarization signal directly reflects lysozyme concentration. Accuracy was validated by spiking known amounts (10 mg and 20 mg) of pure lysozyme standard into tablet samples. Recovery rates of lysozyme demonstrate excellent agreement between its measured and expected amounts according to known requirements [32], indicating minimal matrix effects. Precision, assessed by calculating RSD, also indicates the method’s reproducibility. Future work could involve exploring the method’s applicability to other dosage forms, such as capsules and suspensions, and optimizing the assay for even higher throughput. This rapid, sensitive, and specific FPA method offers a significant improvement for quality control and routine analysis of lysozyme content in tablet formulations and can be extended to other protein drugs including enzymes in the presence of appropriate fluorescently labeled substrate tracers.

## Figures and Tables

**Table 2:** Results of quantitative determination of lysozyme content in tablet forms by FPIA.

Sample	Average Tablet Weight, mg	Lysozyme Content (mg) Per 1 Tablet			Average Value
		200.0 mg *	400.0 mg *	600.0 mg *	
1	201.8	22.3 ± 1.9	22.8 ± 5.4	20.1 ± 3.5	21.7 ± 3.6
2	200.9	20.6 ± 4.9	22.6 ± 3.6	20.9 ± 2.2	21.3 ± 3.6
3	197.1	22.4 ± 5.4	21.3 ± 4.5	21.1 ± 2.3	21.6 ± 4.4
4	200.4	22.8 ± 9.6	21.7 ± 5.8	25.2 ± 7.1	23.2 ± 5.2
5	200.8	22.9 ± 5.3	22.5 ± 0.2	22.5 ± 0.1	22.6 ± 1.8

* Weight of the sample that was subjected to the analytical procedure.

**Table 3:** Study of accuracy of FPIA by the addition method.

Added, mg	Found, mg (*n* = 3)	Recovery, %
0	22.8 ± 1.8	0
10	32.8 ± 2.8	100.2
20	44.0 ± 3.2	106.0

**Table 4:** Study of the accuracy by the recovery test with evaluation of model mixtures.

Lysozyme Content in % of Nominal	Found, mg (*n* = 3)	Found, %
80%	17.7 ± 3.6	98.3
100%	19.6 ± 4.1	98.0
120%	22.7 ± 2.7	100.1

**Table 5:** Intra-assay precision evaluation.

Sample Weight, mg	RSD, %
200.0	10.8
400.0	8.9
600.0	13.7

## Data Availability

The original contributions presented in this study are included in the article. Further inquiries can be directed to the corresponding authors.

## Abbreviations

The following abbreviations are used in this manuscript:

FPA	fluorescence polarization assay
ELISA	enzyme-linked immunosorbent assay
API	active pharmaceutical ingredient
RSD	relative standard deviation
LOD	limit of detection
